# Biological interaction, esthetics, handling, and loss rate of temporary luting cements — a clinical single-blind randomized controlled trial

**DOI:** 10.1007/s00784-024-05804-1

**Published:** 2024-07-13

**Authors:** Elena Günther, Sebastian Hahnel, Annett Schrock, Oliver Schierz, Sophia Wolter

**Affiliations:** 1 Department of Prosthetic Dentistry and Dental Materials Science, Leipzig University, Liebigstraße 12, 04103 Leipzig, Germany; 2 Department of Prosthetic Dentistry, Regensburg University Medical Center, Franz-Josef-Strauß-Allee 11, 93042 Regensburg, Germany; 3 Department of Prosthetic Dentistry and Materials Science, Medical Faculty, University of Rostock, Strempelstraße 13, 18057 Rostock, Germany

**Keywords:** Dental cements, Dental crown, Dental esthetics, Fixed partial denture, Interim cement, Provisional cement

## Abstract

**Objectives:**

To evaluate three temporary luting cements in terms of their restoration loss rates, biological interactions, esthetic properties, and handling characteristics.

**Materials and methods:**

75 adults requiring fixed prosthodontics voluntarily participated in a single-blind, randomized controlled trial. After preparation, temporary restorations were luted with a randomly selected temporary luting cement (either Provicol QM Plus (PQP), Bifix Temp (BT), or Provicol QM Aesthetic (PQA)). Clinical examinations were performed one to two weeks after cementation. The following criteria were evaluated: tooth vitality, percussion, hypersensitivity, gingival bleeding, odor formation, esthetics, cement handling, removability, cleanability, and retention loss. Antagonistic teeth served as controls. Statistical analysis was performed using the paired t-test, one-way ANOVA, Pearson’s chi-square and Fisher’s exact test, where appropriate.

**Results:**

The overall loss rate of temporary restorations was 16.0%, showing no cement-specific differences. Postoperative hypersensitivity occurred in 8% of cases regardless of cement type. Esthetic impairment was reported by 31% of the PQP-fixed restorations, compared with 4.0% and 4.2% of the BT and PQA-bonded restorations. Cement application was reported to be easy in 100% of cases, excess removal in 88–96%, depending on the cement used.

**Conclusions:**

The choice of luting material affects the esthetic appearance of a temporary restoration and should be considered, particularly in restorations in esthetically demanding areas. No significant differences between the cements were identified regarding biocompatibility, handling, and loss rate.

**Clinical relevance:**

Translucent cements can help to reduce color interferences, resulting in a more appealing appearance of the temporary restoration.

## Introduction

With an increasing number of people retaining their natural teeth into old age, there has been a rise in the number of patients opting for fixed restorations such as fixed partial dentures or crowns [1]. As part of this treatment, a temporary restoration is inserted for the duration of the fabrication of the final fixed prosthesis. The temporary counterpart can protect and stabilize the prepared tooth, shape soft tissues, and preserve chewing function and esthetics [2–4]. Temporary cements are used to insert temporary restorations, allowing both adequate retention and easy removal [5]. Other desirable factors include preferably no interaction with biological tissues, satisfactory esthetics, and easy handling. To date, there have been numerous laboratory studies that evaluated the retention force of temporary cements [5–18]. Some in-vitro studies investigated temporary luting agents in terms of their effect on pulp tissue [19–21], and their optical appearance [22, 23]. However, there is a lack of clinical investigations on the performance of temporary cements. A limited number of clinical studies have evaluated the pain perception in patients after tooth preparation and temporary restoration as a function of the temporary cement [24, 25]. There are currently no clinical studies focusing on the loss rates, esthetic appearance, and handling of temporary luting agents.

Temporary luting materials can be categorized according to their setting mechanism (e.g. self-curing/dual curing), their main and side components (e.g. zinc oxide/composite and eugenol/calcium hydroxide), and their shade (e.g. opaque, translucent). Established temporary luting cements are based on zinc oxide and are meanwhile available in eugenol-free formulations. Although eugenol has an antibacterial effect and may prevent hypersensitivity or even pulp necrosis [2, 26, 27], it may also inhibit the polymerization of resin-based materials such as adhesive cements for final restorations [28–32]. In addition, increased elution of eugenol-containing temporary cements has been observed [33, 34]. Eugenol-free temporary cements can be produced on the basis of composite (e.g. Bifix Temp; VOCO GmbH) or zinc oxide and can be combined with calcium hydroxide (e.g. Provicol QM Plus, Provicol QM Aesthetic; VOCO GmbH). Calcium hydroxide may have a protective effect on tooth vitality by promoting the formation of tertiary dentine [35]. Some of these materials are available in a translucent shade (e.g. Bifix Temp, Provicol QM Aesthetic), enabling inconspicuous cementation of temporary restorations. At the same time, temporary cements should be clearly visible and easy to remove. The remaining excess may otherwise lead to inflammation of the gingiva [36, 37], and increased gingival bleeding, which could result in difficulties with subsequent impression taking or final cementation [3]. Moreover, the retention force of temporary cements plays an important role, as low retention strength might result in restoration loss and, consequently, chemical, thermal, or physical damage of dental hard tissue. If the loss of a temporary restoration persists over a longer period of time, possible tooth migration [38] could affect the fit of the final restoration. In order to achieve optimal bonding values and to avoid excessive acids or monomers damaging the dental pulp [4, 39–41], the manufacturer’s mixing ratio must strictly be followed or pre-dosed automix luting materials should be used.

In this context, the aim of the current study was to clinically examine three temporary luting materials with regard to their biological interaction, esthetics, handling, and loss rate. The following null hypotheses were formulated:

1. There is no difference in gingival inflammation between teeth on which temporary restorations were cemented with Bifix Temp (BT), Provicol QM Plus (PQP), and Provicol QM Aesthetic (PQA).
2. There is no difference in hypersensitivity between teeth on which temporary restorations were cemented with BT, PQP, and PQA.
3. There is no difference between the esthetic appearance of BT, PQP, and PQA.
4. There is no difference between the handling of BT, PQP, and PQA.
5. There is no difference between the loss rates of temporary restorations fixed with BT, PQP, and PQA.

## Materials und methods

### Study design

**Fig. 1:**
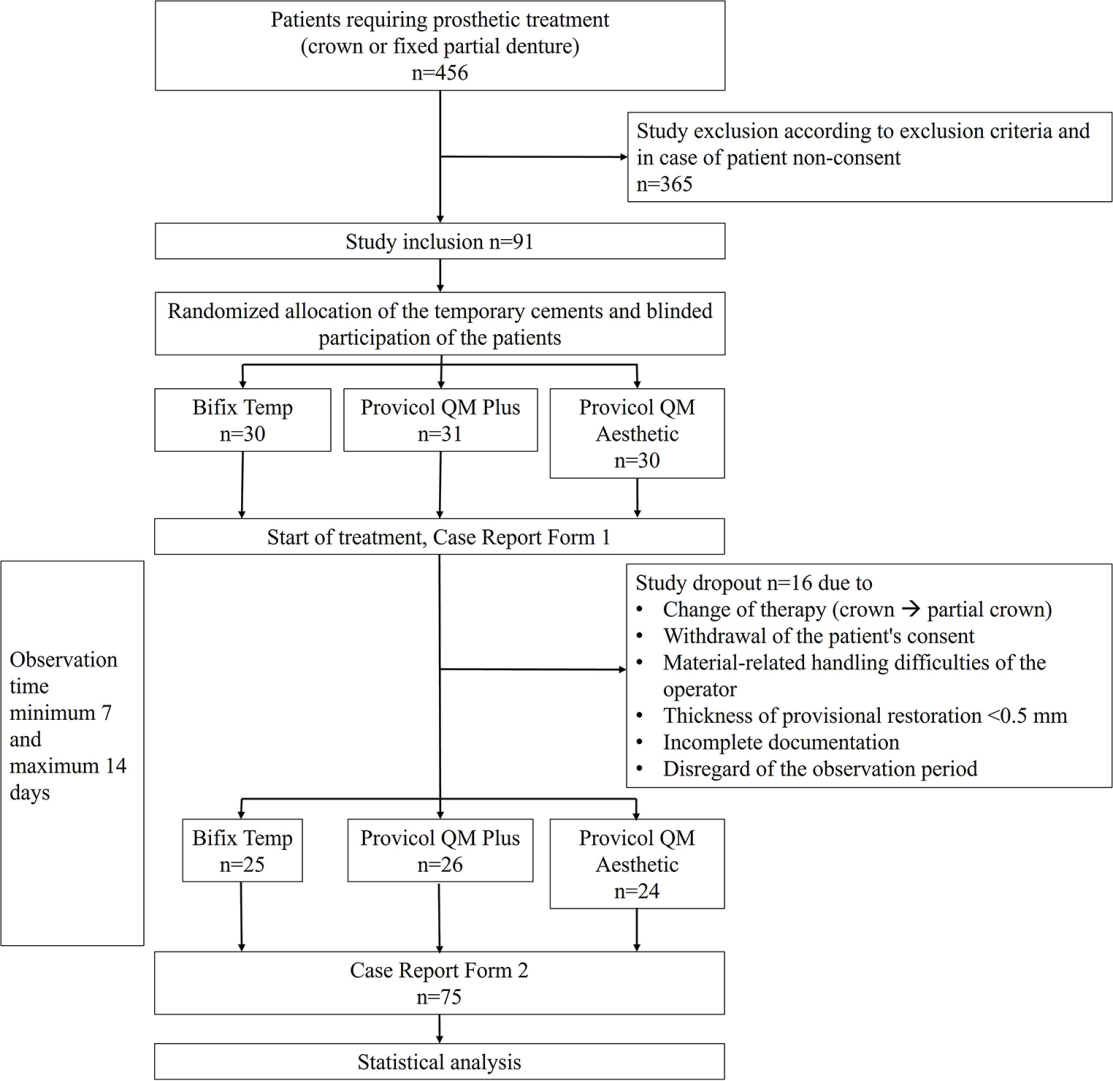
CONSORT flow diagram

The following investigation was conducted as a blinded randomized controlled clinical prospective study. The study project was approved by the Ethics Committee of the Medical Faculty of the University of Leipzig (481/20-ek) and registered in the DRKS (German Register of Clinical Studies, DRKS00024019). The procedures used in this study adhere to the tenets of the Declaration of Helsinki. The subjects were recruited at the Department of Prosthetic Dentistry and Dental Materials Science, Leipzig University, both in the staff treatment and in the undergraduate student courses between April 2021 and August 2023. Certified dentists and undergraduate dental students underwent quarterly training on the correct conduct of the study. The study procedure is shown in Fig. 1.

### Patients

Patients of the University Dental Clinic were screened for voluntary study participation and informed about the trial in verbal and written form. Prior to inclusion, patients provided their written consent to participate in the study. Withdrawing consent was possible at any point without needing to provide an explanation or facing repercussions. To avoid patient bias, one temporary restoration per patient was carried out.

The following inclusion criteria were defined:

- Prosthetic restoration required (crown/fixed partial denture on two abutment teeth).
- Vital study tooth.
- Abutment height at least 3 mm from the preparation margin to the functional inclination [42].
- Voluntary participation.

The following exclusion criteria were defined:

- Known allergy to components of the luting cements.
- Allergy to temporary material.
- Pregnancy.
- Breastfeeding.
- Age less than 18 years.
- Lack of consent.
- Removable dentures to be attached to the temporarily restored study tooth.
- Partial crowns.
- Temporary restoration thickness less than 0.5 mm.

### Materials

A provisional resin-based composite material (Structur 3, VOCO GmbH, Cuxhaven, Germany) was used to fabricate the temporary restoration, which was luted with an automix temporary luting cement randomly selected from three available products (Table 1).

**Table 1:** Ingredients of the temporary luting cements used according to the manufacturer’s instructions (insofar as evident)

Temporary luting material	Abbreviation	Ingredients (base)	Ingredients (catalyst)	Shade
Bifix Temp, VOCO GmbH, Cuxhaven, Germany	BT	urethane dimethacrylate, triethylene glycol dimethacrylate	triethylene glycol dimethacrylate, urethane dimethacrylate, benzoyl peroxide	translucent
Provicol QM Plus, VOCO GmbH, Cuxhaven, Germany	PQP	coconut fatty acid, 2 ethoxybenzoic acid	zinc oxide, calcium hydroxide	opaque
Provicol QM Aesthetic, VOCO GmbH, Cuxhaven, Germany	PQA	coconut fatty acid, 2 ethoxybenzoic acid	zinc oxide, calcium hydroxide	translucent

### Study procedure

For each patient, one vital tooth meeting the inclusion criteria was included in the study. If several teeth were eligible for inclusion, the study tooth was selected clockwise according to an inclusion scheme, starting with the upper last molar on the right side. Prior to preparation, vitality, percussion, and gingival bleeding index of the study tooth and the antagonistic control tooth were recorded in a corresponding questionnaire, known as the Case Report Form (CRF). After tooth preparation, the minimum stump height from the preparation margin to the beginning of the functional inclination was measured. In addition, any existing core fillings of the study tooth were documented. A temporary restoration was then fabricated using a mold or impression (Structur 3, VOCO GmbH). Both temporary and final restoration had to meet the same requirements regarding minimum layer thickness ≥ 0,5 mm, interproximal contacts, marginal fit, occlusion, and polishing. Before placing the temporary restoration, the operator evaluated the minimum layer thickness and subjectively estimated the restoration`s friction (strong, medium, none). Moreover, the luting material was randomly assigned to the blinded patients using a randomization list (block randomization, block size = 10). Subsequent to insertion, handling of the temporary cement was rated by the assistant (simple, complicated), while the operator performed and evaluated the removal of excess material (simple, complicated) and esthetics of the restoration (inconspicuous, conspicuous).

A minimum of 7 and a maximum of 14 days after the preparation, a follow-up session was carried out. A second CRF was used to record whether and on what occasion the temporary restoration was lost due to a lack of retention. In addition, hypersensitivity, biological parameters (gingival bleeding, vitality, percussion) and esthetics of the luting cement were documented. After the temporary restoration’s removal, the operator evaluated its removability (simple, complicated), odor (no, yes), and cleanability of temporary restoration and stump (simple, complicated). The study also noted whether cement residues remained primarily in the temporary restoration, on the tooth, or on the build-up filling.

### Statistical analysis

The data collected were documented in a database (Access 2016, Microsoft) and transferred to Stata (Stata 17.0, Basic Edition, StataCorp LLC, College Station, Texas, USA) for statistical analysis. Gingival differences before and after wearing the temporary restorations were examined using the paired t-test and one-way ANOVA. The Pearson’s chi-square test was applied for sample size calculation, and to analyze group-specific differences with regard to biological interactions, esthetics, manageability, and loss rates. Where appropriate, Fisher’s exact tests were performed after Pearson’s chi-square tests. The level of significance was set to *p* < 0.05.

## Results

### Demographic characteristics

456 patients were screened for potential participation in the study (Fig. 1). After receiving verbal and written information about the study, a total of 91 subjects voluntarily chose to participate. In the course of prosthetic treatment, 16 patients were excluded from the study, resulting in a total of 75 participants with one restoration each eligible for data analysis. On average, the participants were 60 ± 15 years old, with 39 women and 36 men involved (52.0% and 48.0%). Almost half of the treatments were performed in staff treatment sessions and half in undergraduate student courses (44.0% and 56.0%). A total of 33 single temporary crowns (44.0%) and 42 temporary fixed partial dentures (56.0%) were included. The distribution of temporary cements and tooth groups are shown in Table 2.

**Table 2:** Absolute and relative distribution of the temporary restorations with regard to the temporary luting cements

	Bifix Temp *n* (%)	Provicol QM Plus *n* (%)	Provicol QM Aesthetic *n* (%)
**Total number** ***(N = 75)***	25 (33.3)	26 (34.7)	24 (32.0)
Crowns	11 (44.0)	10 (38.5)	12 (50.0)
Fixed partial dentures	14 (56.0)	16 (61.5)	12 (50.0)
**Location**			
Anterior region	9 (36.0)	8 (30.8)	8 (33.3)
Premolar region	10 (40.0)	11 (42.3)	7 (29.2)
Molar region	6 (24.0)	7 (26.9)	9 (37.5)

### Loss rates

During the observation period, a total of twelve temporary restorations (16.0%) lost retention during meals (*n* = 7; 58.4%), oral hygiene measures (*n* = 3; 25%), sleeping (*n* = 1; 8.3%), and other (*n* = 1; 8.3%) (Table 3). No statistically significant differences were identified concerning the loss rate of temporarily cemented crowns and fixed partial dentures (Pearson’s chi-square test, *p* = 0.766; Fisher’s exact = 1.000), tooth groups (Pearson’s chi-square test, *p* = 0.402; Fisher’s exact = 0.591), or the luting material (Pearson’s chi-square test, *p* = 0.612; Fisher’s exact = 0.754).

### Biological interactions

#### Effects on dental tissue

After preparation and temporary restoration, postoperative hypersensitivity occurred in 8% of cases (Table 3). Teeth restored with PQP were less often hypersensitive (3.8%) than teeth from the PQA or BT group (8.3% and 12.0%, respectively). However, this difference was not statistically significant in this study (Pearson’s chi-square test, *p* = 0.585; Fisher’s exact = 0.673). In one case, loss of sensitivity of the abutment tooth was observed.

#### Effects on gingival tissue

The results of the six-point measurement of gingival bleeding before and after temporary restoration are shown in Table 4. No statistically significant differences were identified between the gingival bleeding indices (GBI) of the study and control teeth before treatment (t-test; *p* = 0.214), within the study and control teeth after treatment (t-test; *p* = 0.583 and *p* = 0.289), or between the luting agents. In addition, no statistically significant differences were found in gingival bleeding before and after temporary restoration within crowns (t-test; *p* = 0.244) and fixed partial prostheses (t-test; *p* = 0.773), nor between each other (t-test; *p* = 0.209). In this clinical examination, the restoration location (anterior, premolar, molar) did not influence gingival inflammation (one-way ANOVA; *p* = 0.652).

#### Odor formation

In ten cases (13.3%), a subjectively perceived odor from the temporary restoration was noted after intraoral wearing. There were no statistically significant group differences in this study (Pearson’s chi-square test, *p* = 0.505; Fisher’s exact = 0.523).

**Table 3:** Complications during the observation period

	Bifix Temp	Provicol QM Plus	Provicol QM Aesthetic
**Retention loss of temporary restorations**			
Total n (%)	3 (12.0)	5 (19.2)	4 (16.7)
Crowns n (%)	2 (18.2)	2 (20.0)	2 (16.7)
Fixed partial dentures n (%)	1 (7.1)	3 (18.8)	2 (16.7)
Anterior region n (%)	1 (11.1)	1 (12.5)	1 (12.5)
Premolar region n (%)	1 (10.0)	4 (36.4)	1 (14.3)
Molar region n (%)	1 (16.7)	0 (0.0)	2 (22.2)
Average minimum thickness of temporary restoration (mm)	0.83	0.75	0.93
Average frictional height (mm)	3.3	3.5	3.8
Average number of core-filled surfaces/study tooth	1.7	1.0	3.5
**Average initial friction**			
Strong n (%)	3 (100)	1 (20)	2 (50)
Moderate n (%)	0 (0.0)	3 (60)	2 (50)
None n (%)	0 (0.0)	1 (20)	0 (0.0)
**Frequency of hypersensitivity, loss of sensitivity, and percussion sensitivity of the study teeth**			
Hypersensitivity n (%)	3 (12.0)	1 (3.8)	2 (8.3)
Loss of sensitivity n (%)	1 (4.0)	0 (0.0)	0 (0.0)
Percussion sensitivity n (%)	0 (0.0)	0 (0.0)	0 (0.0)

**Table 4:** Modified gingival bleeding index (GBI) of study and control teeth before and after temporary restoration. Six-point measurement (6 points bleeding = 100%). SD: standard deviation

	Tooth	GBI before temporary cementation (mean/SD, %)	GBI after observation period (mean/SD, %)	*p*-value
**Bifix Temp**	Study tooth	24.2 ± 31.2	25.0 ± 35.5	0.840
	Control tooth	19.7 ± 24.5	16.7 ± 20.6	0.383
**Provicol QM Plus**	Study tooth	15.1 ± 18.9	14.3 ± 21.9	0.833
	Control tooth	15.9 ± 12.3	11.9 ± 15.0	0.171
**Provicol QM Aesthetic**	Study tooth	21.1 ± 21.4	25.4 ± 26.3	0.426
	Control tooth	16.7 ± 22.2	17.5 ± 23.9	0.841

#### Esthetics

**Fig. 2:**
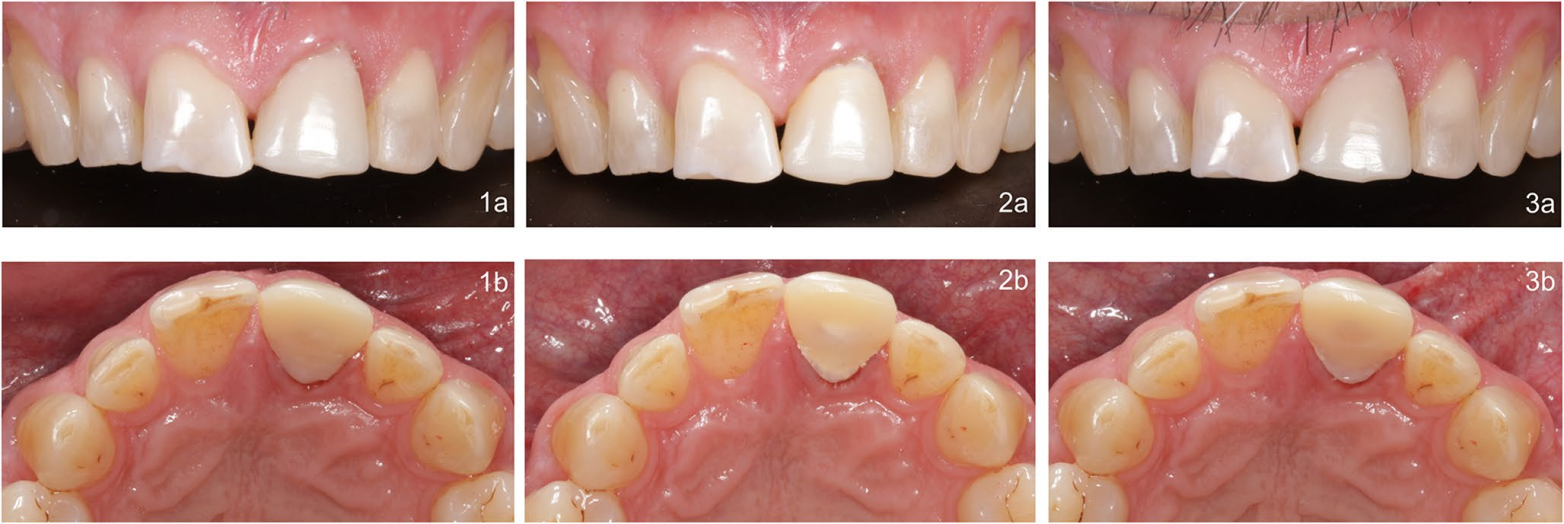
Optical appearance of a temporary crown (Structur 3, VOCO GmbH) on the left upper central incisor luted with Bifix Temp (**1**), Provicol QM Plus (**2**), and Provicol QM Aesthetic (**3**). Frontal view (**a**) and top view (**b**). The temporary crown and the stump were thoroughly cleaned between the cementations (crown: sandblasting, stump: scaler). Compared to BT and PQA, PQP shimmers opaquely through thin areas of the temporary crown (cervical (2**a**) and palatal (2**b**))

At the time of cementation, the appearance of the three cements varied significantly from one another (Pearson’s chi-square test, *p* = 0.005; Fisher’s exact = 0.007). In ten cases, the luting material was described as conspicuous (Table 5). While restorations placed with BT or PQA featured esthetic limitations in only one case each (white shimmering through), eight temporary restorations cemented with PQP were described as esthetically displeasing (opaque). Figure 2 illustrates the appearance of the temporary cements BT, PQP, and PQA. After the wearing period, only three temporarily cemented restorations remained esthetically unpleasant, whereby no significant group differences were identifiable (Pearson’s chi-square test, *p* = 0.381; Fisher’s exact = 0.631). In one case, a yellowish discoloration of the BT luting cement was observed.

**Table 5:** Esthetics of the temporary cements examined before and after intraoral wearing

	Bifix Temp	Provicol QM Plus	Provicol QM Aesthetic
**Esthetics directly after temporary cementation **(***p* = 0.005**)			
Inconspicuous n (%)	24 (96.0)	18 (69.2)	23 (95.8)
Conspicuous n (%)	1 (4.0)	8 (30.8)	1 (4.2)
**Esthetics after observation period** (***p *= 0.381**)			
Inconspicuous n (%)	23 (92.0)	20 (76.9)	19 (79.2)
Conspicuous n (%)	1 (4.0)	2 (7.7)	0 (0.0)
Missing data (e.g. due to retention loss) n (%)	1 (4.0)	4 (15.4)	5 (20.8)

#### Handling

The application of the temporary automix cements using a mixing tip was rated as easy by the assisting colleagues, regardless of the cement group (Table 6). According to practitioners, there were about three times fewer difficulties with excess removal of the dual-curing material BT than of the self-curing materials PQP and PQA. However, this difference was not statistically significant in the current examination (Pearson’s chi-square, *p* = 0.529; Fisher’s exact = 0.616). In most cases, cement residues were predominantly found in the temporary restoration (Table 6). There were no cement-specific differences (Pearson’s chi-square, *p* = 0.585; Fisher’s exact = 0.757).

Temporary restorations luted with BT and PQA were twice less likely to be considered difficult to clean compared to PQP (Table 6). Overall, no statistically significant group-specific differences could be identified for either the cleaning of the temporaries or the cleaning of the abutments (Pearson’s chi-square test, *p* = 0.135 and *p* = 0.404; Fisher’s exact = 0.173 and 1.000).

**Table 6:** Handling of the temporary luting cements

	Bifix Temp	Provicol QM Plus	Provicol QM Aesthetic
**Application (insertion session)**			
Simple n (%)	25 (100)	26 (100)	24 (100)
Complicated n (%)	0 (0.0)	0 (0.0)	0 (0.0)
**Excess removal (insertion session)**			
Simple n (%)	24 (96.0)	23 (88.5)	21 (87.5)
Complicated n (%)	1 (4.0)	3 (11.5)	3 (12.5)
**Location of cement residue**			
In temporary restoration n (%)	22 (88)	22 (84.6)	19 (49.2)
On tooth n (%)	1 (4.0)	0 (0.0)	1 (4.2)
On core-filling n (%)	0 (0.0)	0 (0.0)	0 (0.0)
Missing data (e.g. due to retention loss) n (%)	2 (8.0)	4 (15.4)	4 (16.6)
**Cleanability of the temporary restoration (removal session)**			
Simple n (%)	20 (80.0)	15 (57.7)	18 (75.0)
Complicated n (%)	3 (12.0)	7 (26.9)	2 (8.3)
Missing data (e.g. due to retention loss) n (%)	2 (8.0)	4 (15.4)	4 (16.7)
**Cleanability of the abutment (removal session)**			
Simple n (%)	22 (88.0)	22 (84.6)	19 (79.2)
Complicated n (%)	1 (4.0)	0 (0.0)	0 (0.0)
Missing data (e.g. due to retention loss) n (%)	2 (8.0)	4 (15.4)	5 (20.8)

## Discussion

This clinical study examined three temporary luting cements with regard to their biological interaction, esthetics, handling, and loss rate of the temporary restoration. No significant differences were identified between the three cements in terms of biological interaction (gingival inflammation, hypersensitivity), handling, and loss rates. Consequently, hypotheses one, two, four and five can be accepted. In terms of esthetics, restorations luted with BT and PQA were significantly more likely to be rated as inconspicuous compared to those cemented with PQP; thus, the third hypothesis must be rejected.

Although the clinical demands toward temporary luting materials in terms of biocompatibility, handling, esthetics and retention are high, there have been hardly any research efforts in this regard to date. Until now, temporary luting materials have mainly been investigated in laboratory studies, for example in the context of implant restorations [16, 17], with regard to their influence on subsequent adhesive cementation [31, 32], and in terms of microleakage [33, 34]. Clinical studies focused on temporarily cemented restorations on natural teeth are rare.

Although no statistically significant differences in biological interactions were found in the current clinical study, postoperative hypersensitivity was detected about three times less frequently in the PQP and PQA group than in the BT group. This may be due to PQP and PQA containing calcium hydroxide, which contributes to the formation of tertiary dentin. In the current work, 6.0% of the patients who had been supplied with temporary restorations luted with calcium hydroxide-containing cements reported postoperative hypersensitivities. Willershausen et al. detected postoperative hypersensitivity requiring analgesia in 20.8% of cases using the first-generation Provicol calcium hydroxide cement [25]. The reason for the higher incidence of postoperative hypersensitivity in the former study is unclear. Furthermore, the manufacturer does not provide any detailed information about the exact composition of the ingredients, their concentration, or possible differences between the products. A recent review estimates the incidence of postoperative loss of sensitivity of prepared abutment teeth due to pulp necrosis and periapical pathologies in 5.02% and 3.63% of cases, respectively. The implementation of treatments in undergraduate student courses and the application of temporary restorations over a period of more than two weeks increased the incidence [27]. In this study, which was conducted by undergraduate dental students and certified dentists within seven to fourteen days, the incidence of sensitivity loss was significantly lower at 1.3%. According to animal experiments, cement components that could potentially damage the pulp do not affect the dentin-pulp complex if a minimum dentin thickness of 0.5 mm is respected [21]. It can therefore be assumed that the above-mentioned complications can potentially be attributed to an invasive preparation method. Regarding biological complications such as gingival inflammation, there are no clinical studies analyzing gingival bleeding as a function of temporary cement. In the current clinical trial, the kind of temporary cement, the location, and type of temporary restoration did not influence gingival inflammation. All patients received oral hygiene instructions and, if necessary, professional teeth cleaning prior to prosthetic treatment. However, there was a considerable variation in interindividual oral hygiene abilities, as indicated by the high standard deviation during the observation period (Table 4). These results indicate comparable biological interactions of the tested cements with soft tissue and that oral hygiene ability is not influenced by the temporary restoration. To the best of the authors’ knowledge, no studies have yet examined the handling of temporary cements. Regarding cement application, there has been no differences between the tested materials in the current clinical investigation. This is not surprising as all materials tested were available in pre-dosed automix cartridges, which do not require manual mixing. No statistically significant differences regarding excess removal during the insertion were observed in this trial. However, excess removal of BT was less often rated as complicated compared to PQP and PQA. This may be attributed to the dual-curing BT being easier to remove in one piece than the excess of the self-curing PQP and PQA. The fact that all three cements were predominantly found in the temporary restoration after removal is particularly favorable as minimal manipulation of the tooth stump and gingiva is required for cleaning. This enables a time efficient and gentle procedure that does not impair subsequent treatments such as impression taking or final insertion. In terms of cleaning the temporary restoration from cement residue, more PQP-fixed restorations were deemed as difficult to clean compared to BT and PQA, which may be due to the rather crumbly consistency of the PQP material. Parallel to this clinical study, a laboratory investigation of the temporary cements BT, PQP, and PQA was conducted by the authors’ research group to investigate their microstructural properties, retention strength, and translucency [18]. BT provided significantly higher retention forces compared to the control cement Meron (glass ionomer cement, VOCO GmbH), PQP, and PQA. However, the presumed lower loss rate of temporary restorations fixed with BT could not be confirmed in the current clinical study. As loss rates of temporary restorations have not been investigated before, the overall loss rate of 16.0% for temporary crowns and fixed partial dentures can be used for comparison in future studies. The indifference of loss rates regarding restoration type, location, and luting material suggests that the materials tested are comparable and that the study protocol was rigorously adhered to (e.g. minimum stump height, predominant frictional preparation, minimum thickness of temporary restoration, interproximal contacts). In terms of optical properties, Groß et al. demonstrated that BT exhibited the highest level of translucency in vitro, followed by PQA, Meron, and PQP [18]. Further laboratory studies affirmed that translucent cements such as PQA and BT are less likely to show through temporary material than opaque cements such as Provicol [22, 23]. This clinical study confirms the assumptions from laboratory studies that opaque temporary luting materials contribute to a significantly more conspicuous appearance of the temporary restoration. In contrast, translucent cements seem to facilitate the transmission of optical properties of the abutment tooth and the temporary restoration, contributing to a more natural appearance of the temporary restoration.

One of the limitations of the present study is the long recruitment phase of the patients (28 months), which was partly due to the COVID-19 pandemic and lecture-free periods. In addition, although randomization of the luting cement was performed, it would have been desirable to implement stratified randomization according to the treatment area (anterior teeth, premolars, molars) since different chewing forces are expected on the restorations depending on the intraoral area. For better comparability of retention loss and oral hygiene parameters, future studies should focus on either crowns or fixed partial dentures. A double-blind study design might have helped to reduce potential operator bias. However, due to the indifferences revealed between the materials tested, a relevant bias is unlikely. Additionally, a comparison with temporary cements from other manufacturers would have been interesting and helped with avoiding potential manufacturer bias. As loss rates of temporarily fixed restorations have not been previously described in literature, a thorough sample size calculation was not possible beforehand. Consequently, this investigation provides the necessary data for sample size calculation for future studies.

## Conclusion

In this randomized controlled clinical trial, the choice of the interim cement affected the esthetic appearance of temporary restorations. As biological interaction, handling, and restoration loss rates were similar between the cements, this observation should be taken into account when deciding on clinical application, especially in esthetically demanding areas.

## Data Availability

The dataset supporting the conclusions of this article is included within the article. The datasets used and analyzed during the current study are available from the corresponding author on reasonable request.
